# Climatic forcing and larval dispersal capabilities shape the replenishment of fishes and their habitat‐forming biota on a tropical coral reef

**DOI:** 10.1002/ece3.3779

**Published:** 2018-01-15

**Authors:** Shaun K. Wilson, Martial Depcyznski, Rebecca Fisher, Thomas H. Holmes, Mae M. Noble, Ben T. Radford, Michael Rule, George Shedrawi, Paul Tinkler, Christopher J. Fulton

**Affiliations:** ^1^ Marine Science Program Department of Biodiversity, Conservation and Attractions Kensington WA Australia; ^2^ Oceans Institute University of Western Australia Crawley WA Australia; ^3^ Australian Institute of Marine Science Crawley WA Australia; ^4^ Research School of Biology The Australian National University Canberra ACT Australia; ^5^ Deakin University School of Life and Environmental Sciences Warrnambool Vic. Australia

**Keywords:** climate forcing, larval behavior, nursery habitats, population dynamics

## Abstract

Fluctuations in marine populations often relate to the supply of recruits by oceanic currents. Variation in these currents is typically driven by large‐scale changes in climate, in particular ENSO (El Nino Southern Oscillation). The dependence on large‐scale climatic changes may, however, be modified by early life history traits of marine taxa. Based on eight years of annual surveys, along 150 km of coastline, we examined how ENSO influenced abundance of juvenile fish, coral spat, and canopy‐forming macroalgae. We then investigated what traits make populations of some fish families more reliant on the ENSO relationship than others. Abundance of juvenile fish and coral recruits was generally positively correlated with the Southern Oscillation Index (SOI), higher densities recorded during La Niña years, when the ENSO‐influenced Leeuwin Current is stronger and sea surface temperature higher. The relationship is typically positive and stronger among fish families with shorter pelagic larval durations and stronger swimming abilities. The relationship is also stronger at sites on the coral back reef, although the strongest of all relationships were among the lethrinids (*r* = .9), siganids (*r* = .9), and mullids (*r* = .8), which recruit to macroalgal meadows in the lagoon. ENSO effects on habitat seem to moderate SOI–juvenile abundance relationship. Macroalgal canopies are higher during La Niña years, providing more favorable habitat for juvenile fish and strengthening the SOI effect on juvenile abundance. Conversely, loss of coral following a La Niña‐related heat wave may have compromised postsettlement survival of coral dependent species, weakening the influence of SOI on their abundance. This assessment of ENSO effects on tropical fish and habitat‐forming biota and how it is mediated by functional ecology improves our ability to predict and manage changes in the replenishment of marine populations.

## 1 INTRODUCTION

Most marine plants and animals have a bipartite life history whereby reproductive gametes or larvae spend the early stages of their life in the open ocean. During this phase, larvae may be transported considerable distances by oceanic currents (Wilson, Fulton, et al. 2016). The extent to which currents shape larval transport and distribution patterns is affected by their pelagic larval duration (PLD) and movement behavior (Levin, 2006; Morgan & Fisher, 2010; Shanks, 2009). Both behavior and PLD vary among taxa and are sensitive to climatic factors such as current strength and sea temperature (Figueiredo, Baird, Harii, & Connolly, 2014; Green & Fisher, 2004; O'Connor et al., 2007). Recruitment to marine populations is consequently governed by complex interactions between larval supply, their functional abilities, and climate.

The El Niño Southern Oscillation (ENSO) exerts a powerful influence on the sea temperature and flow of major ocean currents in the tropical Pacific Ocean. This influence may be extended to the adjacent Indian Ocean through the Indonesian Flow Through (Godfrey, 1996). During extreme ENSO events, dramatic shifts in marine climate can cause thermal stress to habitat‐forming biota such as corals and algae in both the Pacific and Indian Oceans, which can lead to extensive mortality of habitat‐forming biota and regime shifts (Graham, Jennings, MacNeil, Mouillot, & Wilson, 2015; Hughes et al., 2017; Wernberg et al., 2016). Annual shifts in ENSO can also be linked to fluctuations in the supply of fish and invertebrate recruits, which may ultimately influence marine populations (Attrill & Power, 2002; Cheal, Delean, Sweatman, & Thompson, 2007).

El Niño conditions in the Indo‐Pacific are typically associated with warmer sea surface temperatures, and along the east coast of Australia recruitment of fish is higher during these years (Cheal et al., 2007), while in French Polynesia recruitment is higher during La Niña, when water temperatures are cooler and oceanic productivity is high (Lo‐Yat et al., 2011). Along the western coast of Australia, ENSO is closely associated with the strength of the southward‐flowing Leeuwin Current (Pearce & Phillips, 1988). During La Niña years, the poleward‐flowing Leeuwin Current is stronger, bringing warm water to the southwest of Australia. Conversely, water temperatures are typically lower along the southwest during El Niño years, when the Leeuwin Current is weaker (Feng, Meyers, Pearce, & Wijffels, 2003). On southwest temperate reefs, Leeuwin Current strength has been correlated with abundance of juvenile lobster and has been used to predict future stocks and manage fisheries (Caputi, 2008; Caputi et al., 2016; Lenanton, Caputi, Kangas, & Craine, 2009). Similarly, abundance of damselfish (Hutchins & Pearce, 1994) and baldchin grouper (Cure, Hobbs, & Harvey, 2015) is high on temperate reefs during La Niña years. There is, however, considerable variation in ENSO–recruitment relationships, with the recruitment of some temperate species being higher during El Niño years (Caputi et al. 1996), although the causes of this variation remain equivocal. Moreover, it is unclear how ENSO‐driven shifts in the Leeuwin Current affect tropical species at the northern extent of the Leeuwin Current.

To investigate how ENSO may affect the size of tropical marine populations, we examined links between the Southern Oscillation Index (SOI) and the abundance of juvenile fishes, coral spat, and the structure of macroalgal meadows along 150 km of the Ningaloo reef ecosystem. Data were collected annually over a period of 8 years, which spanned some of the strongest El Niño and La Niña events on record (Feng, McPhaden, Xie, & Hafner, 2013; Lim, Kovach, Pawson, & Vernieres, 2017). To explore why ENSO influences taxa differently, we also examined how well reproductive and ecological traits in fish explained variation in the strength and direction of the relationship between SOI and juvenile fish abundance. Combined, these assessments of ENSO effects on tropical taxa improve understanding of how large‐scale fluxes in climate influence recruitment strength, advancing our ability to predict and manage future marine populations.

## 2 METHODS

The influence of climate forcing on a suite of tropical marine taxa was investigated by examining the potential relationship between the SOI and the annual abundance of juvenile fishes, coral recruits, and macroalgal meadows within the fringing coral reef ecosystem of Ningaloo Marine Park, Western Australia. The SOI‐juvenile abundance relationship is measured at oceanic scales and is calculated as the difference in surface air pressure between Tahiti and Darwin, with a single value representing the region. Monthly SOI data were obtained from the Australian Bureau of Meteorology http://www.bom.gov.au/climate/current/soihtm1.shtml and an annual SOI calculated as the average for the 6 months preceding field surveys. This time frame incorporates the season when fish become reproductively active and accounts for the time required for larvae to return to the reef.

**Figure 1:**
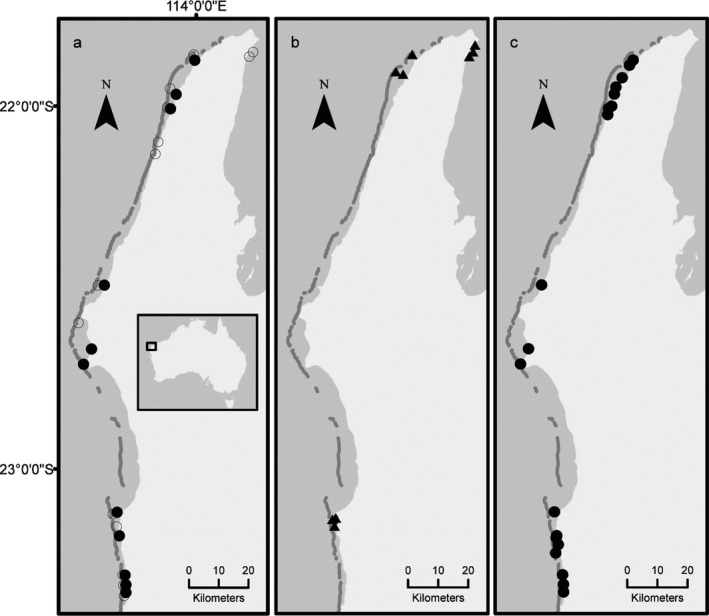
Field sites at Ningaloo Reef in Western Australia: a) fish survey sites in both coral (open circles) and macroalgae habitats (closed circles), b) coral recruitment sites (closed triangles), and c) macroalgae survey sites (closed circles)

Juvenile fish abundance was estimated annually at 15 coral and 11 macroalgal, shallow water (1–5 m) sites, protected from prevailing oceanic swells (Figure 1a). Thirteen of the coral sites were on back reef areas on the western side of North West Cape (W), and two were on sheltered reefs in Exmouth Gulf (G). All macroalgal sites were discrete lagoonal patches (L) on the western side of North West Cape, characterized by canopy‐forming algae, predominantly from the genus *Sargassum*. Surveys were conducted during February–March at each site from 2010 to 2017 (inclusive), which immediately follows the peak recruitment period for fish in this region (McIlwain, 2003). Winter surveys (July) were also carried out from 2013 to 2015 and were used to calculate the extent of recruitment season for each family (further details in Statistical analyses). At each site × year combination, the number of juvenile fish from 11 prominent families were counted within nine 1 × 30 m haphazardly placed transects, separated by at least 5 m. Collectively, these families encompass the vast majority of conspicuous fish diversity found on tropical reefs, as well as important targets for fishing in this region (Ryan et al., 2015). Transects were located haphazardly as this does not require the use of permanent transect markers, which may be hazardous to vessels and allows efficient surveying over the entire site. While there may be increased variation from using random rather than fixed transects, juvenile fish are mobile and we expect this source of variation is small. Indeed, comparisons at different spatial scales have found variation within sites to be small compared to variation among sites at Ningaloo (Fulton et al., 2014). Nonetheless, all transect data were averaged at the site level and sites used as replicates in analyses which reduced any influence of transect variability on results.

Recruitment of coral spat onto settlement tiles was measured annually from 2009 to 2016 at ten coral sites in shallow (1–4 m) water, protected from the oceanic swell (Figure 1b). Three of these sites were in Exmouth Gulf (G) and seven were on the western side of North West Cape (W). At each site, 15–21 terracotta tiles were deployed across three areas (5–7 tiles per area) and the same three areas and tile locations were used each year. Tiles were deployed in January/February and retrieved in April/May, ~1 month after the mass spawning is expected at Ningaloo (Gilmour, Speed, & Babcock, 2016). Each 10 × 10 × 1 cm tile was attached to the reef with a stainless steel bolt and baseplate, raised 2–3 cm above the substrate with a spacer (Mundy, 2000).

The cover, density, and height of canopy‐forming macroalgae were measured each summer (February) at all 11 macroalgal sites surveyed for fish, plus an additional eight sites from 2013 to 2017 (Figure 1a,c). Within each site, macroalgal cover was quantified along six haphazardly placed 10 m line intercept transects, separated by at least 5 m. Holdfast density of canopy‐forming macroalgae was counted within six 0.5 × 0.5 quadrats evenly placed along each 10 m transect. Similarly, canopy height was assessed at six evenly spaced points along the 10 m transect, using a ruler to measure the height of floating thalli above the substratum. Random transects were used over a fixed transect sampling strategy, as this allows efficient surveying over the entire site, increasing the total number of replicate transects able to be assessed within the given logistic constraints. All data were averaged at the site level and sites used as replicates in analyses which reduced any influence of transect or quadrat variability on results.

### 2.1 Statistical analyses

Annual summer averages of juvenile fish abundance, coral recruitment, and macroalgal structure were calculated for data collected at each site and year. Annual averages were then compared to SOI for the corresponding year and Pearson's correlation coefficient calculated to assess the strength and direction of any relationship between SOI and juvenile fish abundance (measured at the family level), coral recruitment and algal structure. As spatial and temporal sampling for fish, macroalgae, and coral recruits differ, these were all modeled independently. Furthermore, as some sites have inherently low recruitment, correlation coefficients were only calculated for sites where fish from the relevant family were observed in four or more of the survey years.

Data for juvenile fish, coral recruitment, and macroalgal structure were also pooled into Gulf, Western back reef and Lagoon habitats and annual means calculated for each of these habitats (Table 1). These annual means were regressed against annual SOI values to assess the influence of climate over larger spatial scales.

**Table 1:** Correlative relationships (*r*) between the Southern Oscillation Index (SOI), juvenile fish, coral recruitment, and macroalgae calculated at (n) number of sites and across all data within a habitat

Habitat	Taxa	Sites			All data		
		*n*	Range of *r*	Mean *r* (*SE*)	*r*	*F*	*p*
Back reef (W)	Acanthuridae	9	−.52 to .84	.29 (.16)	.54	2.52	.164
	**Apogonidae**	13	−.14 to .78	.44 (.07)	**.83**	**13.02**	**.011**
	Chaetodontidae	13	−.50 to .79	.13 (.15)	.30	0.60	.467
	**Labridae**	14	−.02 to .78	.57 (.06)	**.73**	**6.89**	**.039**
	**Monacanthidae**	14	−.16 to .88	.59 (.09)	**.90**	**24.73**	**.003**
	Nemipteridae	9	.69 to .69	.45 (.10)	.51	2.14	.194
	**Pomacentridae**	14	.35 to .84	.68 (.03)	**.80**	**10.70**	**.017**
	Scarinae	14	−.25 to .88	.22 (.07)	.29	0.55	.487
	Scleractinian Corals	7	−.21 to .83	.30 (.14)	.29	2.46	.168
Back reef (G)	Acanthuridae	2	−.13 to .68	.27 (.40)	.66	4.68	.074
	Apogonidae	2	−.08 to .00	−.04 (.04)	.00	0.00	.992
	Chaetodontidae	2	.22 to .63	.42 (.20)	.45	1.51	.266
	Labridae	2	.21 to .45	.33 (.12)	.33	0.72	.429
	Nemipteridae	2	.39 to .45	.42 (.03)	.48	1.82	.226
	Pomacentridae	2	.35 to .56	.46 (.10)	.49	1.94	.213
	Scarinae	2	.02 to .23	.12 (.10)	.15	0.13	.728
	Sclearctinian Corals	3	.05 to .32	.22 (.08)	.21	0.30	.608
Lagoon (L)	Acanthuridae	8	−.67 to .83	−.02 (.19)	−.05	0.01	.912
	Apogonidae	10	−.42 to .83	.12 (.14)	.01	0.00	.981
	Chaetodontidae	8	−.48 to .79	.16 (.17)	.47	1.69	.241
	Labridae	11	−.49 to .54	.24 (.10)	.45	1.52	.264
	**Lethrinidae**	10	.37 to .82	.65 (.04)	**.92**	**32.07**	**.001**
	**Mullidae**	11	.16 to .86	.57 (.07)	**.79**	**10.08**	**.019**
	Pomacentridae	10	−.17 to .83	.28 (.10)	.50	1.99	.208
	Scarinae	11	−.59 to .69	−.16 (.13)	−.39	1.11	.333
	**Siganidae**	4	.24 to .80	.54 (.12)	**.91**	**28.84**	**.002**
	Macroalgal cover	19	−.92 to .88	.02 (.12)	.11	0.04	.862
	Macroalgal density	19	−.73 to .61	.14 (.10)	.33	0.36	.592
	Macroalgal height	19	−.25 to .78	.26 (.07)	.80	5.25	.106

The probability (*p*) of significant relationships between taxa abundance and SOI was tested across all data using ANOVA with F statistic.

Only taxa where correlation coefficients were detected in two or more sites within a habitat are presented. Bold text and values highlight where significant (*p* < .05) relationships exist between SOI and taxa abundance when all site data are pooled within a habitat. W, Western coast back reef, L, Western Coast Lagoon, G, Gulf back reef sites.

Spatial and taxonomic differences in the strength of the relationship between juvenile fish abundance and SOI were investigated using permutational analysis of variance (PERMANOVA) with 9,999 permutations (Anderson, Gorley, & Clarke, 2008) using Primer 7.0.8. Fish families were entered as fixed factors, habitats (Gulf, Western back reef, and Lagoon) as random factors, and the correlation coefficient estimated within sites were replicates. PERMANOVA was carried out on a resemblance matrix based on Euclidean distance and any significant differences further investigated with pairwise PERMANOVA.

The influence of family‐level reproductive and ecological traits on the strength of the relationship between SOI and juvenile fish abundance was investigated using generalized additive mixed models (GAMM) using a full‐subset analysis (Table 2). Pearson's correlation coefficient calculated at the site level for each family was modeled as the response variable and site was included as a random factor. Other explanatory variables considered in the analysis were swimming ability, the length of time larval fish that are expected to be in the open ocean (PLD), the extent of the recruitment season, and spawning mode (broadcast or demersal). As a measure of swimming ability, we used reported critical swimming speeds (U‐crit) of late‐stage larval fish captured in light traps or crest nests (Table 3). U‐crit measures aerobic swimming endurance of fish and is based on swimming flume experiments where speed is increased step‐wise over time until a maximum speed is obtained for each individual fish (Fisher & Leis, 2010; Plaut, 2001). U‐crit swimming speed has been shown to be strongly related to larval routine activity, as well as in situ and sustained swimming speeds (Fisher & Leis, 2010; Fulton, 2010). The time larvae spend in the open ocean can be calculated from settlement marks on otoliths or larvae raised in captivity (Selkoe & Toonen, 2011) and is referred to as the pelagic larval duration (PLD). Both U‐crit information and PLD information were taken from the literature and used to compute family‐level averages (based on reported mean values for individual species) for our analyses (Table 3). Family‐level averages were required because information across all trait variables was not available at finer taxonomic resolution. The length of the recruitment season was calculated by dividing the number of fish in each family observed during the winter to the number observed in the summer using data collected from 2013 to 2015. All possible combinations of three or less explanatory variables were considered in these analyses, and the best combination of variables selected based on Akaike information criteria corrected for small sample size (AICc). Model weights were also calculated and summed for each predictor, and used to assess which reproductive and ecological variables had the strongest relationship with the correlation coefficient (Burnham & Anderson, 2003). All GAMM were carried out in R (version 3.3.1, R core development team).

**Table 2:** Best models for explaining variance between the Southern Oscillation Index (SOI) and juvenile fish

Model	ΔAICc	wAICc	*R* ^2^	*df*
PLD	0	0.244	.061	2
PLD, U‐crit, Habitat	0.927	0.153	.153	6.09
PLD, U‐crit	1.277	0.129	.092	3.78
Spawning	1.972	0.091	.041	2
U‐crit	2.252	0.079	.065	3.88
Spawning, U‐crit × Spawning	2.852	0.059	.126	7.12
Habitat, U‐crit × Habitat	2.909	0.057	.154	7.42
PLD, Habitat	3.115	0.051	.119	5.61
U‐crit, Habitat	4.337	0.028	.106	5.76
U‐crit, Spawning	4.692	0.023	.058	3

Only the 10 best models from a full‐subset analysis are displayed. Model selection is based on lowest delta Akaike information criteria corrected for small sample size (ΔAICc) and model weights (wAICc). Explanatory variables considered were: pelagic larval duration (PLD), and critical swimming speed (U‐crit), which were sourced from the literature (Table 3) as well as spawning mode (Spawning: demersal or broadcast), length of the recruitment season (Season), and Habitat (Gulf or western back reef and lagoon).

**Table 3:** Explanatory variables used in generalized additive mixed‐model, full‐subset analyses

Family	PLD ± *SE* ^reference^	Season	U‐crit ± *SE* ^reference^	Spawning
Acanthuridae	62.7 ± 4.5^1,2,3^	0.10	46.8 ± 9.8^22,23^	Broadcast
Apogonidae	22.5 ± 0.9^1,4,5^	0.01	20.2 ± 7.4^22,23,24,25^	Demersal
Chaetodontidae	38.0 ± 2.7^1,3^	0.36	39.2 ± 12.6^22,23^	Broadcast
Labridae	39.3 ± 1.7^1,3,4,6,7,8,9,10,11^	0.37	12.8 ± 3.2^23,26^	Broadcast
Lethrinidae	33.5 ± 5.9^1,3,12,13^	0.02	38.4 ± 14.5^22,24^	Broadcast
Monacanthidae	24.0 ± 4.0^1^	0.00	25.3 ± 13.7^22,23^	Demersal
Mullidae	43.7 ± 4.5^3,14^	0.11	47.0^22^	Broadcast
Nemipteridae	19.0^1^	0.02	34.3 ± 9.9^22^	Broadcast
Pomacentridae	22.4 ± 0.4^1,3,9,10,11,15,16,17,18,19^	0.27	35.6 ± 13.0^22,23,24,25^	Demersal
Scarinae	44.7 ± 2.7^1,4^	0.26	6.0^23^	Broadcast
Siganidae	32.5 ± 2.5^20,21^	0.00	67.1 ± 21.7^22^	Broadcast

Average and standard error (*SE*), pelagic larval duration (PLD), and critical swimming speed (U‐crit) calculated from values in the literature. Reproductive season (Season) is the number of fish observed in each family during the winter relative to the number observed in the summer over a 3‐year period (2013–2015). Spawning mode (Spawning) information came from Fishbase (Froese and Pauly 2017). PLD data sourced from: 1. (Brothers, Williams, & Sale, 1983) 2. (McCormick, 1999) 3. (Wilson & McCormick, 1999) 4. (Ishihara & Tachihara, 2011) 5. (Job & Bellwood, 2000) 6. (Caselle, 1997) 7. (Colin, 1982) 8. (Sponaugle & Cowen, 1997) 9. (Victor & Wellington, 2000) 10. (Wellington & Robertson, 2001) 11. (Wellington & Victor, 1992) 12. (Leis, Sweatman, & Reader, 1996) 13. (Leis & Carson‐Ewart, 1997) 14. (McCormick, 1994) 15. (Brothers & Thresher, 1985) 16. (Kerrigan, 1996) 17. (Stobutzki, 1998) 18. (Thresher, Colin, & Bell, 1989) 19. (Wilson & McCormick, 1997) 20. (Bryan & Madraisau, 1977) 21. (May, Popper, & McVey, 1974). U‐crit data sourced from: 22. (Fisher, Leis, Clark, & Wilson, 2005) 23. (Hogan, Fisher, & Nolan, 2007) 24. (Fisher & Wilson, 2004) 25. (Bellwood & Fisher, 2001) 26. (Leis, Hay, & Gaither, 2011).

## 3 RESULTS

**Figure 2:**
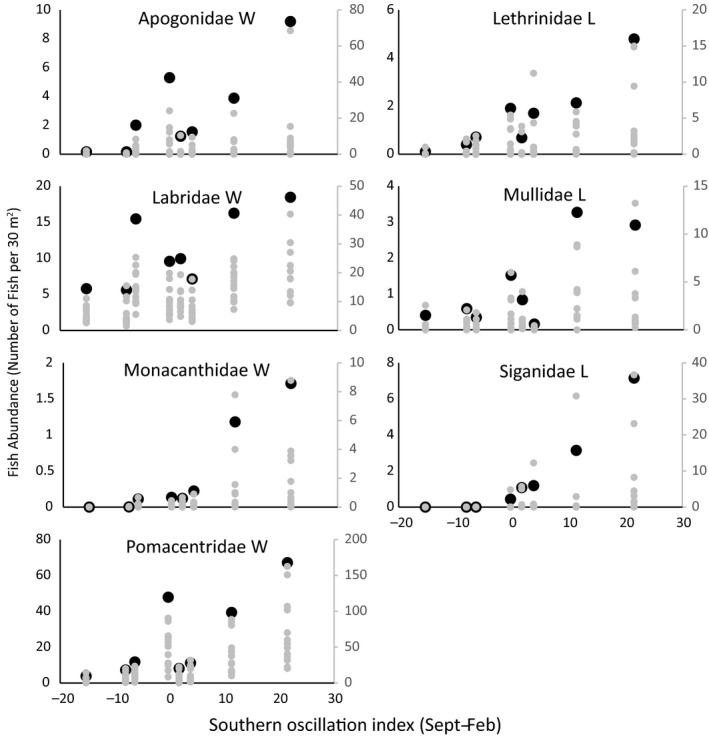
Influence of the Southern Oscillation Index (SOI) on juvenile fish abundance in coral reef and macroalgal meadows at Ningaloo. Only families with significant (*p* < .05) relationships with SOI when data are pooled at the habitat level are shown. Large black dots represent juvenile abundance averaged across all sites within a habitat (Western back reef W, Lagoon L; left *y*‐axis), and small gray dots are site‐level values (right *y*‐axis)

Climatic conditions, indicated by the SOI, generally had a strong effect on the annual abundance of juvenile fishes across Ningaloo, with more juvenile fish being recorded during high SOI (La Niña) years (Table 1). On the more exposed western coast, this relationship was generally weaker in the algae‐dominated lagoon in comparison with the back reef coral habitat (*F*_2,189_ = 13.23, *p* < 0.001), although the strength of the relationship between SOI and juvenile fish abundance also varied among families (*F*_10,189_ = 7.54, *p* < 0.001). Strong positive relationships with SOI were evident within the Lethrinidae, Mullidae, and Siganidae (Table 1, Figure 2). Fish from these three families were predominantly found at sites within the lagoon, although there were also strong positive relationships between SOI and the abundance of juvenile apogonids, labrids, monacanthids, and pomacentrids at the Western back reef sites (Table 1, Figure 2). Notably, we found no significant relationships between SOI and either fish or coral recruits within the Gulf region of Ningaloo.

**Figure 3:**
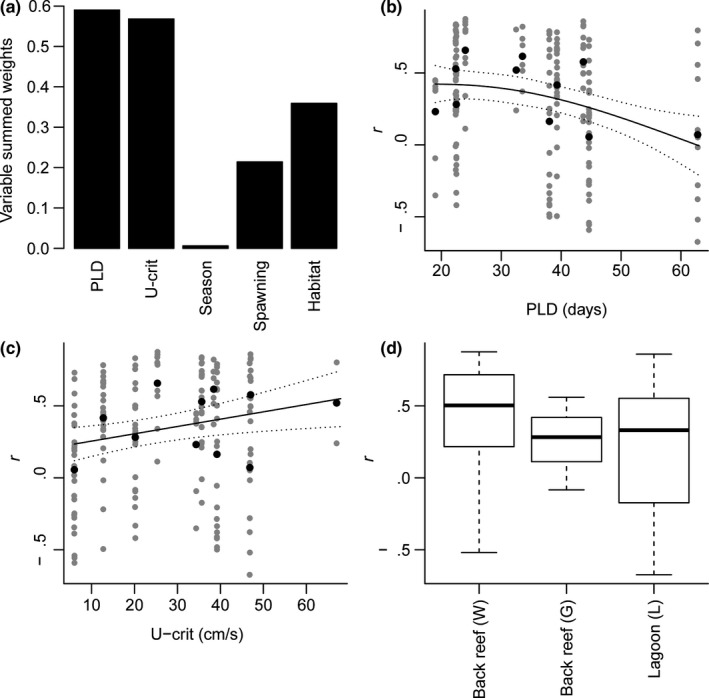
Relative importance of: pelagic larval duration (PLD), critical swimming speed (U‐crit), spawning mode (Spawning), length of the recruitment season (Season) and Habitat on the strength of the relationships between the Southern Oscillation Index (SOI) and juvenile fish abundance (a). The influence of pelagic larval duration (PLD) (b), critical swimming speed (U‐crit, c) and habitat: Western back reef (W), Lagoon (L), and Gulf back reef (G) (d), on the correlation coefficient (*r*) of the SOI, juvenile fish abundance relationship for 173 site x family combinations. Models and 95% confidence intervals (dotted lines) for PLD and U‐crit relationships are based on site‐level (gray dots) information and family‐level averages are shown as black dots. Boxes from box and whisker plots display second and third quartiles, and the dark line is the median correlation coefficient for each habitat

Estimates of pelagic larval duration (PLD) were the best at explaining family‐level differences in the relationships between SOI and juvenile fish abundance (Table 2, Figure 3a). The relationship was typically positive when PLD was <30 days, and was more likely to be negative as PLD increased (Figure 3b). However, the specific influence of PLD on these relationships was weak, with both spawning mode and swimming ability explaining similar levels of variation in SOI–juvenile fish abundance relationships. Demersal spawning fish had a higher correlation coefficient than pelagic spawning families, suggesting the positive relationship between SOI and juvenile abundance is stronger among demersal spawners. Likewise, the SOI abundance relationships were strong and positive among fast‐swimming fish juveniles (high U‐crit), while correlation coefficients generally declined with decreases in family‐level U‐crit (Figure 3c). Habitat also had a weak influence on the SOI abundance relationship (Figure 3a), fish on the western back reef having stronger positive coefficients compared to gulf and lagoon habitats (Figure 3d). There was, however, widespread variation within habitats, with some weak negative relationships at both back reef and macroalgal lagoon sites.

Annual coral recruitment also tended to be higher during La Niña years within both the Gulf and Western regions of Ningaloo, although there was considerable variation in the direction and strength of this relationship among sites (Table 1). Similarly, the summer height, density, and cover of canopy‐forming macroalgae within the lagoon were usually higher in La Niña than El Niño years. Summer SOI explained up to 60% of the variance in macroalgal meadow height, although relationships between SOI and other aspects of macroalgal structure were generally weak, especially when measured at the site level.

## 4 DISCUSSION

Critical processes such as gamete production, recruitment, and growth in fish populations are closely aligned with climatic fluctuations, and the ENSO appears to be a key metric in understanding and measuring this climatic forcing on population demographics across a range of tropical and temperate marine taxa (Ong et al., 2016). We show that juvenile fish abundance, coral recruitment, and the canopy height of macroalgal beds are all positively affected by SOI, although there is considerable variation in the strength of this relationship according to both taxa‐specific and spatial factors.

The relationship between SOI and juvenile fish abundance is slightly stronger on the western back reef than the lagoon, especially among pomacentrids, labrids, and apogonids, many of which are closely associated with corals as juveniles (Coker, Wilson, & Pratchett, 2014; Wilson et al., 2010). The only strong relationships in the lagoon are among those taxa that are predominantly found in macroalgal habitats as juveniles and may be considered macroalgal specialists (Wilson et al., 2010, 2017). Indeed, these are among the strongest of all SOI–fish abundance relationships and the strength of the relationship in the lagoon may be partially attributable to macroalgal habitat also being related to SOI.

We found that canopy height of macroalgae in the lagoon was generally higher during warmer La Niña years, when fish recruitment was also strongest. Sea surface temperature is known to influence macroalgal growth and standing biomass (Hwang, Tsai, & Lee, 2004), and in tropical Sargassaceae, canopy biomass and height are seasonally linked to sea surface temperature, with higher canopy biomass during the warmer summer months (Fulton et al., 2014). A positive correlation between SOI and canopy height suggest this seasonal growth may be driven by extension of existing macroalgal thalli rather than increased densities of new algae. Importantly canopy height is closely related to abundance of both adult and juvenile fish (Evans, Wilson, Field, & Moore, 2014; Levin & Hay, 1996; Lim, Wilson, Holmes, Noble, & Fulton, 2016), suggesting taller canopies during La Niña may provide exceptional habitat for macroalgal specialists and greater survival of fish recruits. Similar findings have been recorded in Mexico, where recruitment of grouper is linked to *Sargassum* availability and ENSO (Aburto‐Oropeza, Sala, Paredes, Mendoza, & Ballesteros, 2007). This emphasizes the importance of macroalgal habitat on the survival of juvenile fish and their capacity to contribute to future adult populations (Lim et al., 2016; Wilson et al., 2017).

Fish with strong positive SOI abundance relationships in the lagoon are also strong swimmers and may hone in on macroalgae as a settlement cue (Arvedlund & Takemura, 2006). Strong swimming may allow these fish to access and navigate lagoonal areas behind the back reef to locate suitable macroalgal habitat patches. Strong swimming may also facilitate movement to suitable macroalgal patches in the winter, following the seasonal breakdown of canopy structure (Lim et al., 2016; Wilson et al., 2014).

The influence of life history and ecological variables on the strength and direction of the SOI‐juvenile abundance relationship was generally weak, suggesting these variables alone are insufficient to predict how ENSO affects the recruitment rates of different taxa. We found that the relationship between the SOI and abundance of juvenile fishes was marginally stronger in families with shorter larval durations. Larval fish that spend a shorter amount of time in the pelagic environment tend to have shorter dispersal distances and levels of connectivity, although there is considerable variation in these relationships (Bay, Crozier, & Caley, 2006; Selkoe & Toonen, 2011; Shanks, 2009). Stronger currents during La Niña years may facilitate recruitment of fish that have a limited time window to find appropriate settlement habitats and metamorphose into their juvenile form. Additionally, warmer water can promote greater reproductive output, faster growth, and higher survival of larvae (Pankhurst & Munday, 2011). These factors may help to explain why recruitment is generally higher in La Niña years. However, family differences in PLD had only a weak effect on the strength of the SOI abundance relationship and some juveniles with relatively long PLD's were more abundant during La Niña years. Similarly, spawning mode and swimming ability explained <10% of the variation in the SOI abundance relationship among fish families. Explanatory power of these traits may be improved by examining this effect among species rather than families, although our results suggest factors such as habitat availability and quality may be more important in determining why the SOI abundance relationship differs among fish.

Compared to macroalgal fields, the composition and cover of coral communities usually remain stable between seasons and disturbances that cause dramatic changes have been irregular at Ningaloo (Speed et al., 2013). When there is extensive change in coral cover, for example after bleaching, recovery is slow and this condition may persist for several years (Graham, Nash, & Kool, 2011). This could explain the relatively poor relationship between SOI and coral recruitment at Ningaloo, as a La Niña‐related heat wave in 2011 caused extensive bleaching and mortality of large coral colonies in the Gulf (Depczynski et al., 2013). Reduced occurrence of large, mature colonies reduces local supply of coral recruits (Gilmour, Smith, Heyward, Baird, & Pratchett, 2013) and compromises the SOI recruitment relationship. Reduced coral cover in the Gulf may have also contributed to weaker relationships between SOI and juvenile fish compared to sites on western back reefs. Changes to coral cover can have a profound effect on fish recruitment (Feary, Almany, McCormick, & Jones, 2007), which may alter adult populations and diversity of fish communities for considerable lengths of time (Graham et al., 2007; Jones, McCormick, Srinivasan, & Eagle, 2004). Moreover, temporal recruitment trends in the Gulf and western back reef sites differ (Feng, Colberg, Slawinski, Berry, & Babcock, 2016; Wilson, Depczynski, et al. 2016), suggesting inconsistent influence of SOI on currents in these two areas.

Understanding the influence of large‐scale climatic events on the abundance of marine species is crucial for disentangling the effects of natural and anthropogenic drivers of change. Such distinctions allow a systematic assessment of human influence on the environment and are the basis of informed management decisions. The predominantly positive effect that La Niña events have on recruitment of fish and corals demonstrates that ENSO has a strong effect on fish, corals, and algae at Ningaloo, and emphasizes the role this cycle has on populations globally. The effect is likely to be particularly strong in locations where prominent boundary currents drive productivity. At Ningaloo, it appears the links between the Leeuwin Current and the Indonesian Flow Through provide the mechanism for these strong links between SOI and coral and fish recruitment.

Critically, the influence of ENSO on recruitment patterns clearly differs among taxa. Accordingly, the ability to predict future populations and interpret long‐term trends in abundance is dependent on the strength of the SOI abundance relationship. We find this relationship is particularly robust among fish that have strong habitat affiliations, especially when that habitat is also routinely rather than irregularly influenced by ENSO. Confidence in predicting the influence of ENSO on future populations of these fish should be high, although local abundance will also be affected by the immediate availability of suitable habitat. Given the mounting evidence that major ocean currents are shifting in their strength of flow (Wilson, Fulton, et al. 2016), these forcing effects must be considered in adaptive management responses to marine climate change.

## DATA ACCESSIBILITY

Data used in analyses are housed at the Department of Biodiversity, Conservation and Attractions, Western Australia, and can be accessed via email MarineDataRequests@dbca.wa.gov.au.

## CONFLICT OF INTEREST

None declared.

## AUTHORS' CONTRIBUTIONS

SKW, MD, BTR, THH, and CJF conceived the study, SKW, MD, THH, MMN, MR, PT, GS, and CJF performed the research, SKW, RF, and BTR analyzed data, and all authors contributed to writing the paper.

## References

[ece33779-bib-0001] Aburto‐Oropeza, O. , Sala, E. , Paredes, G. , Mendoza, A. , & Ballesteros, E. (2007). Predictability of reef fish recruitment in a highly variable nursery habitat. Ecology, 88, 2220–2228. https://doi.org/10.1890/06-0857.1 17918400 10.1890/06-0857.1

[ece33779-bib-0002] Anderson, M. , Gorley, R. N. , & Clarke, R. K. (2008). Permanova+ for primer: Guide to software and statistical methods. PRIMER‐E: Plymouth, UK.

[ece33779-bib-0003] Arvedlund, M. , & Takemura, A. (2006). The importance of chemical environmental cues for juvenile *Lethrinus nebulosus* Forsskal (Lethrinidae, Teleostei) when settling into their first benthic habitat. Journal of Experimental Marine Biology and Ecology, 338, 112–122. https://doi.org/10.1016/j.jembe.2006.07.001

[ece33779-bib-0004] Attrill, M. J. , & Power, M. (2002). Climatic influence on a marine fish assemblage. Nature, 417, 275–278. https://doi.org/10.1038/417275a 12015600 10.1038/417275a

[ece33779-bib-0005] Bay, L. K. , Crozier, R. H. , & Caley, M. J. (2006). The relationship between population genetic structure and pelagic larval duration in coral reef fishes on the Great Barrier Reef. Marine Biology, 149, 1247–1256. https://doi.org/10.1007/s00227-006-0276-6

[ece33779-bib-0006] Bellwood, D. R. , & Fisher, R. (2001). Relative swimming speeds in reef fish larvae. Marine Ecology Progress Series, 211, 299–303. https://doi.org/10.3354/meps211299

[ece33779-bib-0007] Brothers, E. B. , & Thresher, R. E. (1985). Pelagic duration, dispersal, and the distribution of Indo‐Pacific coral reef fishes. The Ecology of Coral Reefs, 3, 53–69.

[ece33779-bib-0008] Brothers, E. B. , Williams, D. M. B. , & Sale, P. F. (1983). Length of larval life in twelve families of fishes at ‘One Tree Lagoon’, Great Barrier Reef, Australia. Marine Biology, 76, 319–324. https://doi.org/10.1007/BF00393035

[ece33779-bib-0009] Bryan, P. G. , & Madraisau, B. B. (1977). Larval rearing and development of *Siganus lineatus* (Pisces: Siganidae) from hatching through metamorphosis. Aquaculture, 10, 243–252. https://doi.org/10.1016/0044-8486(77)90005-9

[ece33779-bib-0010] Burnham, K. P. , & Anderson, D. R. (2003). Model selection and multimodel inference: A practical information‐theoretic approach. Berlin, Germany: Springer Science & Business Media.

[ece33779-bib-0011] Caputi, N. (2008). Impact of the Leeuwin Current on the spatial distribution of the puerulus settlement of the western rock lobster (*Panulirus cygnus*) and implications for the fishery of Western Australia. Fisheries Oceanography, 17, 147–152. https://doi.org/10.1111/j.1365-2419.2008.00471.x

[ece33779-bib-0500] Caputi, N. , Fletcher, W. J. , Pearce, A. , Chubb, C. F. (1996). Effect of the Leeuwin Current on the Recruitment of Fish and Invertebrates along the Western Australian Coast. Marine and Freshwater Research, 47, 147–155.

[ece33779-bib-0012] Caputi, N. , Kangas, M. , Denham, A. , Feng, M. , Pearce, A. , Hetzel, Y. , & Chandrapavan, A. (2016). Management adaptation of invertebrate fisheries to an extreme marine heat wave event at a global warming hot spot. Ecology and Evolution, 6, 3583–3593. https://doi.org/10.1002/ece3.2137 28725352 10.1002/ece3.2137 PMC5513294

[ece33779-bib-0013] Caselle, J. E. (1997). Recruitment and early post‐settlement processes in coral reef fishes. Santa Barbara: University of California.

[ece33779-bib-0014] Cheal, A. J. , Delean, S. , Sweatman, H. , & Thompson, A. A. (2007). Spatial synchrony in coral reef fish populations and the influence of climate. Ecology, 88, 158–169. https://doi.org/10.1890/0012-9658(2007)88[158:SSICRF]2.0.CO;2 17489464 10.1890/0012-9658(2007)88[158:ssicrf]2.0.co;2

[ece33779-bib-0015] Coker, D. J. , Wilson, S. K. , & Pratchett, M. S. (2014). Importance of live coral habitat for reef fishes. Reviews in Fish Biology and Fisheries, 24, 89–126. https://doi.org/10.1007/s11160-013-9319-5

[ece33779-bib-0016] Colin, P. L. (1982). Spawning and larval development of the hogfish, Lachnolaimus maximus. Fishery Bulletin 80: 853–862.

[ece33779-bib-0017] Cure, K. , Hobbs, J.‐P. A. , & Harvey, E. S. (2015). High recruitment associated with increased sea temperatures towards the southern range edge of a Western Australian endemic reef fish *Choerodon rubescens* (family Labridae). Environmental Biology of Fishes, 98, 1059–1067. https://doi.org/10.1007/s10641-014-0339-3

[ece33779-bib-0018] Depczynski, M. , Gilmour, J. P. , Ridgway, T. , Barnes, H. , Heyward, A. J. , Holmes, T. H. , … Wilson, S. K. (2013). Bleaching, coral mortality and subsequent survivorship on a West Australian fringing reef. Coral Reefs, 32, 233–238. https://doi.org/10.1007/s00338-012-0974-0

[ece33779-bib-0019] Evans, R. D. , Wilson, S. K. , Field, S. N. , & Moore, J. A. Y. (2014). Importance of macroalgal fields as coral reef fish nursery habitat in north‐west Australia. Marine Biology, 161, 599–607. https://doi.org/10.1007/s00227-013-2362-x

[ece33779-bib-0020] Feary, D. A. , Almany, G. R. , McCormick, M. I. , & Jones, G. P. (2007). Habitat choice, recruitment and the response of coral reef fishes to coral degradation. Oecologia, 153, 727–737. https://doi.org/10.1007/s00442-007-0773-4 17566781 10.1007/s00442-007-0773-4

[ece33779-bib-0021] Feng, M. , Colberg, F. , Slawinski, D. , Berry, O. , & Babcock, R. (2016). Ocean circulation drives heterogeneous recruitments and connectivity among coral populations on the North West Shelf of Australia. Journal of Marine Systems, 164, 1–12. https://doi.org/10.1016/j.jmarsys.2016.08.001

[ece33779-bib-0022] Feng, M. , McPhaden, M. J. , Xie, S.‐P. , & Hafner, J. (2013). La Niña forces unprecedented Leeuwin Current warming in 2011. Scientific Reports, 3, 1277 https://doi.org/10.1038/srep01277 23429502 10.1038/srep01277 PMC3572450

[ece33779-bib-0023] Feng, M. , Meyers, G. , Pearce, A. , & Wijffels, S. (2003). Annual and interannual variations of the Leeuwin Current at 32°S. Journal of Geophysical Research: Oceans, 108, 3355 https://doi.org/10.1029/2002JC001763

[ece33779-bib-0024] Figueiredo, J. , Baird, A. H. , Harii, S. , & Connolly, S. R. (2014). Increased local retention of reef coral larvae as a result of ocean warming. Nature Climate Change, 4, 498–502. https://doi.org/10.1038/nclimate2210

[ece33779-bib-0025] Fisher, R. , & Leis, J. M. (2010). Swimming speeds in larval fishes: From escaping predators to the potential for long distance migration In Domenici P., & Kapoor B. (Eds.), Fish locomotion: An eco‐ethological perspective (pp. 333–373). USA: CRC Press LLC https://doi.org/10.1201/b10190

[ece33779-bib-0026] Fisher, R. , Leis, J. M. , Clark, D. L. , & Wilson, S. K. (2005). Critical swimming speeds of late‐stage coral reef fish larvae: Variation within species, among species and between locations. Marine Biology, 147, 1201–1212. https://doi.org/10.1007/s00227-005-0001-x

[ece33779-bib-0027] Fisher, R. , & Wilson, S. K. (2004). Maximum sustainable swimming speeds of late‐stage larvae of nine species of reef fishes. Journal of Experimental Marine Biology and Ecology, 312, 171–186. https://doi.org/10.1016/j.jembe.2004.06.009

[ece33779-bib-0501] Froese, R. , & Pauly, D. (2017). FishBase. World Wide Web electronic publication. www.fishbase.org, version (10/2017).

[ece33779-bib-0028] Fulton, C. J. (2010). The role of swimming in reef fish ecology In Domenici P., & Kapoor B. (Eds.), Fish locomotion: An eco‐ethological perspective (pp. 374–406). Enfield, UK: Science Publishers https://doi.org/10.1201/b10190

[ece33779-bib-0029] Fulton, C. J. , Depczynski, M. , Holmes, T. H. , Noble, M. M. , Radford, B. , Wernberg, T. , & Wilson, S. K. (2014). Sea temperature shapes seasonal fluctuations in seaweed biomass within the Ningaloo coral reef ecosystem. Limnology and Oceanography, 59, 156–166. https://doi.org/10.4319/lo.2014.59.1.0156

[ece33779-bib-0030] Gilmour, J. P. , Smith, L. D. , Heyward, A. J. , Baird, A. H. , & Pratchett, M. S. (2013). Recovery of an isolated coral reef system following severe disturbance. Science, 340, 69–71. https://doi.org/10.1126/science.1232310 23559247 10.1126/science.1232310

[ece33779-bib-0031] Gilmour, J. , Speed, C. W. , & Babcock, R. (2016). Coral reproduction in Western Australia. PeerJ, 4, e2010 https://doi.org/10.7717/peerj.2010 27231651 10.7717/peerj.2010 PMC4878369

[ece33779-bib-0032] Godfrey, J. S. (1996). The effect of the Indonesian throughflow on ocean circulation and heat exchange with the atmosphere: A review. Journal of Geophysical Research: Oceans, 101, 12217–12237. https://doi.org/10.1029/95JC03860

[ece33779-bib-0033] Graham, N. A. J. , Jennings, S. , MacNeil, M. A. , Mouillot, D. , & Wilson, S. K. (2015). Predicting climate‐driven regime shifts versus rebound potential in coral reefs. Nature, 518, 94–97. https://doi.org/10.1038/nature14140 25607371 10.1038/nature14140

[ece33779-bib-0034] Graham, N. A. , Nash, K. L. , & Kool, J. T. (2011). Coral reef recovery dynamics in a changing world. Coral Reefs, 30, 283–294. https://doi.org/10.1007/s00338-010-0717-z

[ece33779-bib-0035] Graham, N. A. J. , Wilson, S. K. , Jennings, S. , Polunin, N. V. C. , Robinson, J. , Bijoux, J. P. , & Daw, T. M. (2007). Lag effects in the impacts of mass coral bleaching on coral reef fish, fisheries, and ecosystems. Conservation Biology, 21, 1291–1300. https://doi.org/10.1111/j.1523-1739.2007.00754.x 17883494 10.1111/j.1523-1739.2007.00754.x

[ece33779-bib-0036] Green, B. S. , & Fisher, R. (2004). Temperature influences swimming speed, growth and larval duration in coral reef fish larvae. Journal of Experimental Marine Biology and Ecology, 299, 115–132. https://doi.org/10.1016/j.jembe.2003.09.001

[ece33779-bib-0037] Hogan, J. D. , Fisher, R. , & Nolan, C. (2007). Critical swimming speed of settlement‐stage coral reef fishes from the Caribbean: A methodological and geographical comparison. Bulletin of Marine Science, 80, 219–231.

[ece33779-bib-0038] Hughes, T. P. , Kerry, J. T. , Álvarez‐Noriega, M. , Álvarez‐Romero, J. G. , Anderson, K. D. , Baird, A. H. , … Bridge, T. C. (2017). Global warming and recurrent mass bleaching of corals. Nature, 543, 373–377. https://doi.org/10.1038/nature21707 28300113 10.1038/nature21707

[ece33779-bib-0039] Hutchins, J. B. , & Pearce, A. F. (1994). Influence of the Leeuwin current on recruitment of tropical reef fishes at Rottnest Island, Western Australia. Bulletin of Marine Science, 54, 245–255.

[ece33779-bib-0040] Hwang, R.‐L. , Tsai, C.‐C. , & Lee, T.‐M. (2004). Assessment of temperature and nutrient limitation on seasonal dynamics among species of sargassum from a coral reef in Southern Taiwan. Journal of Phycology, 40, 463–473. https://doi.org/10.1111/j.1529-8817.2004.03086.x

[ece33779-bib-0041] Ishihara, T. , & Tachihara, K. (2011). Pelagic larval duration and settlement size of Apogonidae, Labridae, Scaridae, and Tripterygiidae species in a coral lagoon of Okinawa Island, Southern Japan. Pacific Science, 65, 87–93. https://doi.org/10.2984/65.1.087

[ece33779-bib-0042] Job, S. D. , & Bellwood, D. R. (2000). Light sensitivity in larval fishes: Implications for vertical zonation in the pelagic zone. Limnology and Oceanography, 45, 362–371. https://doi.org/10.4319/lo.2000.45.2.0362

[ece33779-bib-0043] Jones, G. P. , McCormick, M. I. , Srinivasan, M. , & Eagle, J. V. (2004). Coral decline threatens fish biodiversity in marine reserves. Proceedings of the National Academy of Sciences of the United States of America, 101, 8251 https://doi.org/10.1073/pnas.0401277101 15150414 10.1073/pnas.0401277101 PMC419589

[ece33779-bib-0044] Kerrigan, B. A. (1996). Temporal patterns in size and condition at settlement in two tropical reef fishes (Pomacentridae: Pomacentrus amboinensis and *P. nagasakiensis*). Marine Ecology Progress Series, 135, 27–41. https://doi.org/10.3354/meps135027

[ece33779-bib-0045] Leis, J. , & Carson‐Ewart, B. (1997). In situ swimming speeds of the late pelagic larvae of some Indo‐Pacific coral‐reef fishes. Marine Ecology Progress Series, 159, 165–174. https://doi.org/10.3354/meps159165

[ece33779-bib-0046] Leis, J. M. , Hay, A. C. , & Gaither, M. R. (2011). Swimming ability and its rapid decrease at settlement in wrasse larvae (Teleostei: Labridae). Marine Biology, 158, 1239–1246. https://doi.org/10.1007/s00227-011-1644-4

[ece33779-bib-0047] Leis, J. M. , Sweatman, H. P. , & Reader, S. E. (1996). What the pelagic stages of coral reef fishes are doing out in blue water: Daytime field observations of larval behavioural capabilities. Marine and Freshwater Research, 47, 401–411. https://doi.org/10.1071/MF9960401

[ece33779-bib-0048] Lenanton, R. C. , Caputi, N. , Kangas, M. , & Craine, M. (2009). The ongoing influence of the Leeuwin Current on economically important fish and invertebrates off temperate Western Australia–has it changed. Journal of the Royal Society of Western Australia, 92, 111–127.

[ece33779-bib-0049] Levin, L. A. (2006). Recent progress in understanding larval dispersal: New directions and digressions. Integrative and Comparative Biology, 46, 282–297. https://doi.org/10.1093/icb/icj024 21672742 10.1093/icb/icj024

[ece33779-bib-0050] Levin, P. S. , & Hay, M. E. (1996). Responses of temperate reef fishes to alterations in algal structure and species composition. Marine Ecology Progress Series, 134, 37–47. https://doi.org/10.3354/meps134037

[ece33779-bib-0051] Lim, Y.‐K. , Kovach, R. M. , Pawson, S. , & Vernieres, G. (2017). The 2015/16 El Niño event in context of the MERRA‐2 reanalysis: A comparison of the tropical Pacific with 1982/83 and 1997/98. Journal of Climate, 30, 4819–4842. https://doi.org/10.1175/JCLI-D-16-0800.1 10.1175/JCLI-D-16-0800.1 PMC6021759 29962660

[ece33779-bib-0052] Lim, I. E. , Wilson, S. K. , Holmes, T. H. , Noble, M. M. , & Fulton, C. J. (2016). Specialization within a shifting habitat mosaic underpins the seasonal abundance of a tropical fish. Ecosphere, 7, e01212.

[ece33779-bib-0053] Lo‐Yat, A. , Simpson, S. D. , Meekan, M. , Lecchini, D. , Martinez, E. , & Galzin, R. (2011). Extreme climatic events reduce ocean productivity and larval supply in a tropical reef ecosystem. Global Change Biology, 17, 1695–702. https://doi.org/10.1111/j.1365-2486.2010.02355.x

[ece33779-bib-0054] May, R. C. , Popper, D. , & McVey, J. P. (1974). Rearing and larval development of *Siganus canaliculatus* (Park) (Pisces: Siganidae). Micronesica, 10, 285–298.

[ece33779-bib-0055] McCormick, M. I. (1994). Variability in age and size at settlement of the tropical goatfish *Upeneus tragula* (Mullidae) in the northern Great Barrier Reef lagoon. Marine Ecology Progress Series, 103, 1–15. https://doi.org/10.3354/meps103001

[ece33779-bib-0056] McCormick, M. I. (1999). Delayed metamorphosis of a tropical reef fish (*Acanthurus triostegus*): A field experiment. Marine Ecology Progress Series, 176, 25–38. https://doi.org/10.3354/meps176025

[ece33779-bib-0057] McIlwain, J. L. (2003). Fine‐scale temporal and spatial patterns of larval supply to a fringing reef in Western Australia. Marine Ecology Progress Series, 252, 207–222. https://doi.org/10.3354/meps252207

[ece33779-bib-0058] Morgan, S. G. , & Fisher, J. L. (2010). Larval behavior regulates nearshore retention and offshore migration in an upwelling shadow and along the open coast. Marine Ecology Progress Series, 404, 109–126. https://doi.org/10.3354/meps08476

[ece33779-bib-0059] Mundy, C. N. (2000). An appraisal of methods used in coral recruitment studies. Coral Reefs, 19, 124–131. https://doi.org/10.1007/s003380000081

[ece33779-bib-0060] O'Connor, M. I. , Bruno, J. F. , Gaines, S. D. , Halpern, B. S. , Lester, S. E. , Kinlan, B. P. , & Weiss, J. M. (2007). Temperature control of larval dispersal and the implications for marine ecology, evolution, and conservation. Proceedings of the National Academy of Sciences, 104, 1266–1271. https://doi.org/10.1073/pnas.0603422104 10.1073/pnas.0603422104 PMC1764863 17213327

[ece33779-bib-0061] Ong, J. J. L. , Rountrey, A. N. , Zinke, J. , Meeuwig, J. J. , Grierson, P. F. , O'Donnell, A. J. , … Meekan, M. G. (2016). Evidence for climate‐driven synchrony of marine and terrestrial ecosystems in northwest Australia. Global Change Biology, 22, 2776–2786. https://doi.org/10.1111/gcb.13239 26970074 10.1111/gcb.13239

[ece33779-bib-0062] Pankhurst, N. W. , & Munday, P. L. (2011). Effects of climate change on fish reproduction and early life history stages. Marine and Freshwater Research, 62, 1015–1026. https://doi.org/10.1071/MF10269

[ece33779-bib-0063] Pearce, A. F. , & Phillips, B. F. (1988). ENSO events, the Leeuwin Current, and larval recruitment of the western rock lobster. Journal du Conseil: ICES Journal of Marine Science, 45, 13–21. https://doi.org/10.1093/icesjms/45.1.13

[ece33779-bib-0064] Plaut, I. (2001). Critical swimming speed: Its ecological relevance. Comparative Biochemistry and Physiology Part A: Molecular & Integrative Physiology, 131, 41–50. https://doi.org/10.1016/S1095-6433(01)00462-7 10.1016/s1095-6433(01)00462-7 11733165

[ece33779-bib-0065] Ryan, K. , Hall, N. , Lai, E. , Smallwood, C. , Taylor, S. , Wise, B. , &Western Australia & Department of Fisheries (2015). State‐wide survey of recreational boat‐based fishing in Western Australia 2013/14. North Beach, WA: Department of Fisheries, Western Australia.

[ece33779-bib-0066] Selkoe, K. A. , & Toonen, R. J. (2011). Marine connectivity: A new look at pelagic larval duration and genetic metrics of dispersal. Marine Ecology Progress Series, 436, 291–305. https://doi.org/10.3354/meps09238

[ece33779-bib-0067] Shanks, A. L. (2009). Pelagic larval duration and dispersal distance revisited. The Biological Bulletin, 216, 373–385. https://doi.org/10.1086/BBLv216n3p373 19556601 10.1086/BBLv216n3p373

[ece33779-bib-0068] Speed, C. W. , Babcock, R. C. , Bancroft, K. P. , Beckley, L. E. , Bellchambers, L. M. , Depczynski, M. , … Wilson, S. K. (2013). Dynamic stability of coral reefs on the West Australian Coast (ed I Álvarez). PLoS ONE, 8, e69863 https://doi.org/10.1371/journal.pone.0069863 23922829 10.1371/journal.pone.0069863 PMC3726730

[ece33779-bib-0069] Sponaugle, S. , & Cowen, R. K. (1997). Early life history traits and recruitment patterns of Caribbean Wrasses (Labridae). Ecological Monographs, 67, 177–202. https://doi.org/10.1890/0012-9615(1997)067[0177:ELHTAR]2.0.CO;2

[ece33779-bib-0070] Stobutzki, I. C. (1998). Interspecific variation in sustained swimming ability of late pelagic stage reef fish from two families (Pomacentridae and Chaetodontidae). Coral Reefs, 17, 111–119. https://doi.org/10.1007/s003380050104

[ece33779-bib-0071] Thresher, R. E. , Colin, P. L. , & Bell, L. J. (1989). Planktonic duration, distribution and population structure of western and central Pacific damselfishes (Pomacentridae). Copeia, 1989, 420–434. https://doi.org/10.2307/1445439

[ece33779-bib-0072] Victor, B. C. , & Wellington, G. M. (2000). Endemism and the pelagic larval duration of reef fishes in the eastern Pacific Ocean. Marine Ecology Progress Series, 205, 241–248. https://doi.org/10.3354/meps205241

[ece33779-bib-0073] Wellington, G. M. , & Robertson, D. R. (2001). Variation in larval life‐history traits among reef fishes across the Isthmus of Panama. Marine Biology, 138, 11–22. https://doi.org/10.1007/s002270000449

[ece33779-bib-0074] Wellington, G. M. , & Victor, B. C. (1992). Regional differences in duration of the planktonic larval stage of reef fishes in the eastern Pacific Ocean. Marine Biology, 113, 491–498. https://doi.org/10.1007/BF00349176

[ece33779-bib-0075] Wernberg, T. , Bennett, S. , Babcock, R. C. , de Bettignies, T. , Cure, K. , Depczynski, M. , … Wilson, S. (2016). Climate‐driven regime shift of a temperate marine ecosystem. Science, 353, 169–172. https://doi.org/10.1126/science.aad8745 27387951 10.1126/science.aad8745

[ece33779-bib-0076] Wilson, S. K. , Depczynski, M. , Fisher, R. , Holmes, T. H. , O'Leary, R. A. , & Tinkler, P. (2010). Habitat associations of juvenile fish at Ningaloo Reef, Western Australia: The importance of coral and algae. PLoS ONE, 5, e15185 https://doi.org/10.1371/journal.pone.0015185 21151875 10.1371/journal.pone.0015185 PMC2998428

[ece33779-bib-0077] Wilson, S. K. , Depczynski, M. , Fulton, C. J. , Holmes, T. H. , Radford, B. T. , & Tinkler, P. (2016). Influence of nursery microhabitats on the future abundance of a coral reef fish. Proceedings of the Royal Society B: Biological Sciences, 283, 20160903 https://doi.org/10.1098/rspb.2016.0903 27534954 10.1098/rspb.2016.0903 PMC5013763

[ece33779-bib-0078] Wilson, S. K. , Depczynski, M. , Holmes, T. H. , Noble, M. M. , Radford, B. T. , Tinkler, P. , & Fulton, C. J. (2017). Climatic conditions and nursery habitat quality provide indicators of reef fish recruitment strength. Limnology and Oceanography, 62, 1868–1880. https://doi.org/10.1002/lno.v62.5

[ece33779-bib-0079] Wilson, S. K. , Fulton, C. J. , Depczynski, M. , Holmes, T. H. , Noble, M. M. , Radford, B. , & Tinkler, P. (2014). Seasonal changes in habitat structure underpin shifts in macroalgae‐associated tropical fish communities. Marine Biology, 161, 2597–2607. https://doi.org/10.1007/s00227-014-2531-6

[ece33779-bib-0080] Wilson, L. J. , Fulton, C. J. , Hogg, A. M. , Joyce, K. E. , Radford, B. T. M. , & Fraser, C. I. (2016). Climate‐driven changes to ocean circulation and their inferred impacts on marine dispersal patterns. Global Ecology and Biogeography, 25, 923–939. https://doi.org/10.1111/geb.12456

[ece33779-bib-0081] Wilson, D. T. , & McCormick, M. I. (1997). Spatial and temporal validation of settlement‐marks in the otoliths of tropical reef fishes. Marine Ecology Progress Series, 153, 259–271. https://doi.org/10.3354/meps153259

[ece33779-bib-0082] Wilson, D. T. , & McCormick, M. I. (1999). Microstructure of settlement‐marks in the otoliths of tropical reef fishes. Marine Biology, 134, 29–41. https://doi.org/10.1007/s002270050522

